# Iron Deficiency: A Silent Threat in Patients With Heart Failure With Reduced Ejection Fraction

**DOI:** 10.7759/cureus.53542

**Published:** 2024-02-04

**Authors:** Nitin Sarate, Rahul Sonawane, Vinayak Pai, Shifa Karatela, Alhad Mulkalwar

**Affiliations:** 1 Department of Medicine, Seth Gordhandas Sunderdas Medical College and King Edward Memorial Hospital, Mumbai, IND; 2 Department of Medicine, Medical College Baroda and Sir Sayajirao General Hospital, Vadodara, IND; 3 Department of Pharmacology, Dr. D. Y. Patil Medical College, Hospital & Research Centre, Pune, IND

**Keywords:** functional iron deficiency, absolute iron deficiency, nyha dyspnea, anemia, hfref, iron deficiency

## Abstract

Background

Iron deficiency is a prevalent and clinically significant comorbidity in patients with heart failure with reduced ejection fraction (HFrEF). Despite its high prevalence, its impact on clinical outcomes, mortality, and various physiological parameters remains a subject of ongoing investigation. The findings of this study are anticipated to contribute valuable insights into the management and prognosis of patients with HFrEF, potentially informing future interventions and improving patient outcomes. This study aimed to assess the clinical profile of iron deficiency and its implications on morbidity and mortality in patients with HFrEF.

Methodology

A prospective cohort study was conducted at King Edward Memorial Hospital, India, involving 371 patients with HFrEF. Participants underwent comprehensive clinical and laboratory assessments, evaluating iron deficiency with signs, symptoms, comorbidities, dyspnea, elevated jugular venous pressure (JVP), past medical history, and various hematological and biochemical parameters.

Results

Overall, 50% of HFrEF participants were iron deficient (n = 185), of whom 80% (n = 148) had anemia against 43% (n = 81) anemics in iron repletes (n = 186). Of the 185 iron-deficient patients, 44 (11.86%) had absolute iron deficiency and 141 (38%) had functional iron deficiency. Iron deficiency significantly correlated with increased mortality in HFrEF patients (χ^2^ (1, N = 371) = 3.88, p = 0.048). A large positive correlation was observed between absolute iron deficiency and dyspnea severity (r^2^ = 0.949, p = 0.026). Statistically significant differences were found in hemoglobin (anemia), serum iron, serum ferritin, total iron-binding capacity, and transferrin saturation between iron-deficient and iron-replete patients (p < 0.05). However, no statistically significant difference in left ventricular ejection fraction between iron-deficient and replete patients was noted.

Conclusions

Iron deficiency emerges as more than a mere comorbidity in heart failure, becoming a prognostic factor with multifaceted outcomes. Its impact extends beyond cardiovascular consequences, encompassing adverse manifestations such as anemia, ascites, edema, dyspnea, elevated JVP, and a heightened risk of mortality. This intricate interplay positions iron deficiency as a critical determinant, significantly influencing the clinical trajectory and outcomes for patients with HFrEF.

## Introduction

Heart failure is a clinical syndrome characterized by a constellation of symptoms (dyspnea, orthopnea, lower limb swelling) and signs (elevated jugular venous pressure (JVP), pulmonary congestion) often caused by a structural and/or functional cardiac abnormality resulting in reduced cardiac output and/or elevated intracardiac pressures [1]. Although estimates vary depending on the study population, the prevalence of HF is approximately 1-2% and rises to >10% among people over the age of 70 years [2]. This figure may underestimate the true scale of the disease as the estimated prevalence of those with asymptomatic left ventricular (LV) systolic dysfunction in individuals aged over 65 years is 5.5% [3].

The definition of iron deficiency in heart failure differs from that of the general population because heart failure is a chronic inflammatory disease. Iron deficiency in heart failure is defined as a ferritin level of less than 100 ng/mL (absolute iron deficiency) or between 100 and 299 ng/mL and transferrin saturation (TSAT) of less than 20% (functional iron deficiency) [4]. Definitive testing should currently be avoided in the setting of acute heart failure exacerbation because of the variation of ferritin and TSAT [5]. Absolute iron deficiency is reduced or absent iron storage in the bone marrow, liver, and spleen. Functional iron deficiency is normal or increased total body iron stores which are unavailable for incorporation into erythroid precursors for erythropoiesis [6]. Anemia is the consequence of iron deficiency, but we need to stress iron deficiency as its own clinical condition that requires screening and treatment because of its impact on quality of life (QoL) and clinical outcomes.

Iron has an essential role in cardiac physiological processes, including oxygen transport, oxygen storage, oxidative metabolism, lipid, DNA, and RNA metabolism, as well as muscular oxidative metabolism. The hepcidin/ferroportin axis controls systemic iron homeostasis. Hepcidin is the hormone that controls systemic iron availability through the binding of ferroportin. Ferroportin is an iron export protein that releases iron into the circulation from its storage [7]. Hepcidin production is stimulated by inflammation and suppressed by hypoxia. Hepcidin levels, as well as serum soluble transferrin receptor levels, have been proposed as possible markers of iron deficiency, with the latter known to have prognostic value in chronic heart failure [8]. Iron deficiency likely stems from this in combination with absolute causes such as gastrointestinal losses, poor nutrition, and malabsorption [4]. Iron deficiency had previously been thought to have clinical consequences only in the setting of anemia. Iron deficiency without anemia, however, is a significant contributor to increased mortality and hospitalization for heart failure [4]. Despite this, iron deficiency is often underdiagnosed and undertreated. Iron deficiency has an estimated prevalence of 30-79%, but patients are commonly overlooked even to undergo screening [9]. Iron deficiency in heart failure with reduced ejection fraction (HFrEF) has been more extensively studied and is more prevalent in women with advanced New York Heart Association (NYHA) class, those with a higher plasma N-terminal pro-B-type natriuretic peptide (NT-proBNP), and those with higher serum C-reactive protein (CRP) [10]. HFrEF is defined as ejection fraction (EF) less than or equal to 40% and occurs in approximately 46% of those hospitalized with heart failure [11]. Non-anemic iron-deficient patients have worse clinical outcomes than anemic iron-replaced patients, suggesting iron deficiency affects the heart directly and differently than anemia alone. Recently, Rineau et al. [12] demonstrated that iron deficiency without anemia is associated with decreased exercise capacity and reduced left ventricular ejection fraction (LVEF) in mice as well as reduced mitochondrial complex I. These abnormalities are reversed when iron is replaced intravenously, which may explain why treatment is beneficial [13]. Trials investigating skeletal muscle energetics such as FERRIC-HF II have shown that a total repletion dose of iron isomaltoside given at a single sitting was associated with faster skeletal muscle demonstrating better mitochondrial function. These underline the possible importance of skeletal changes despite very little change in hemoglobin [14].

While the association between iron deficiency and adverse outcomes in HFrEF is acknowledged, its prevalence remains unknown, primarily due to infrequent screening for iron deficiency in non-anemic patients, particularly in acute settings. This study can have significant implications for future research focusing on the management and prognosis of iron-deficient individuals with HFrEF. Furthermore, it can pave the way for detailed investigations into the mechanisms underlying this association.

## Materials and methods

A prospective cohort study was conducted at King Edward Memorial Hospital, a tertiary care hospital in western India, with approval from the Institutional Ethics Committee (approval number: EC/133/2021). The study spanned 1.5 years and included 371 participants meeting specific inclusion and exclusion criteria. The sample size was calculated using Cochran’s formula with a prevalence of 54% [15]. Inclusion criteria comprised patients suffering from HFrEF <40%, aged >12 years, willing to participate, and providing written informed consent. Exclusion criteria included patients on iron therapy, blood transfusions, chronic kidney disease (CKD), thalassemia, hemolytic and blood loss anemias, and those unwilling to participate.

A comprehensive assessment, with detailed history and examinations, was documented on a predesigned proforma. Investigations, such as complete blood count (CBC), serum iron studies, liver function tests (LFTs), thyroid function test (TFT), renal function test (RFT), blood glucose tests including fasting and random blood glucose tests, fasting lipid profile, CRP, electrocardiography, and two-dimensional (2D) echocardiography done by the treating physician were noted. No investigations were done for the study. Treatment given by the treating physician was noted.

The collected data were compiled using Microsoft Excel 2010 and analyzed with SPSS version 23 (IBM Corp., Armonk, NY, USA). Descriptive analysis involved expressing numerical data (CBC, LFT, RFT, TFT, and blood sugar levels) as means and standard deviation (SD), while categorical data (age group, gender, comorbidities, body mass index (BMI), clinical features, dyspnea NYHA class, types of iron deficiency (functional iron deficiency and absolute iron deficiency), outcome, 2D echocardiography) were described using numbers and percentages. A comparison between types of Iron deficiency (absolute and functional) and hemodynamic parameters (CBC, LFT, RFT, TFT, and blood sugar parameters) was performed using the Student’s unpaired t-test. The association between iron deficiency and age group, gender, clinical findings, comorbidities, outcome, LVEF, etc., was analyzed using the chi-square test/Fisher’s exact test for categorical variables. Spearman correlation was used to assess the relation between NYHA classes of dyspnea and iron deficiency and its types. A two-tailed p-value <0.05 was considered significant.

## Results

**Figure 1:**
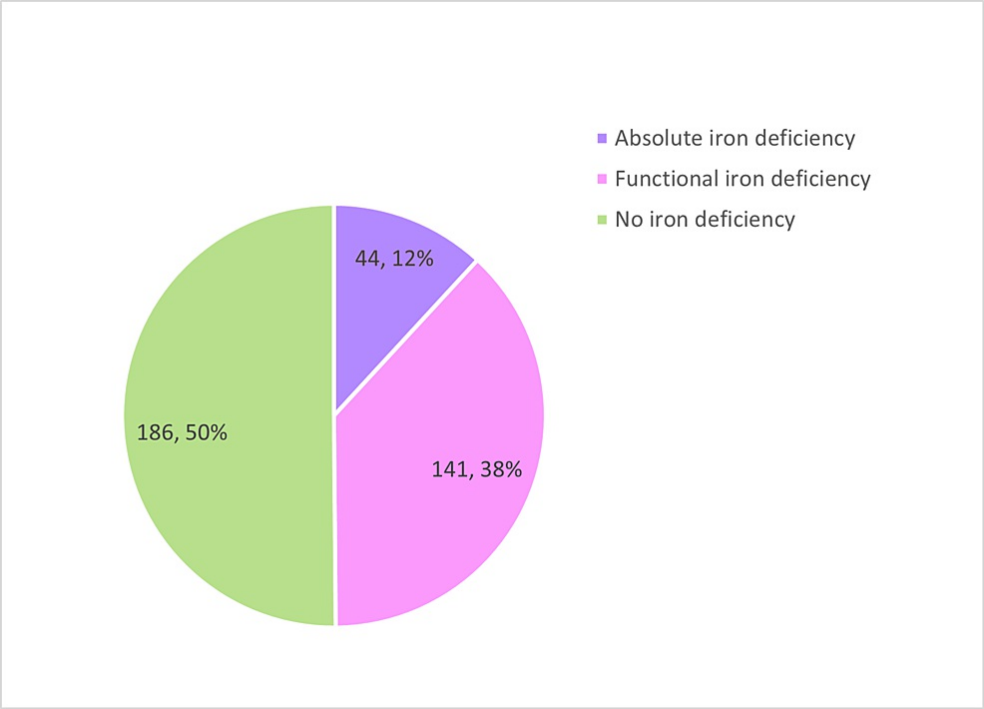
Distribution of HFrEF patients according to iron deficiency. HFrEF = heart failure with reduced ejection fraction

Of the 371 study participants, 44 (11.86%) suffered from absolute iron deficiency and 141 (38%) from functional iron deficiency. Overall, 185 (49.86%) participants had iron deficiency, and 186 (50.14%) participants did not, as shown in Figure 1.

Tables 1-3 show age, gender, and BMI distribution in HFrEF patients, respectively. The participants in the study were aged 23 to 89 years. The average age was 57.53 (±11.85) years. The majority of participants, 157 (42.32%), were in the age group of >60 years. Of the 371 participants, 255 (68.73%) were male, and 116 (31.27%) were female. Of the 185 participants with iron deficiency, the average BMI was 29.10 ± 4.35 kg/m^2^. Of the 44 participants with absolute iron deficiency, the average BMI was 27.20 ± 3.45 kg/m^2^, and of the 141 participants with functional iron deficiency, the average BMI was 28.39 ± 4.05 kg/m^2^. No significant association of BMI was found with absolute, functional, or total iron deficiency (p > 0.05).

**Table 1:** Age distribution in HFrEF patients. HFrEF = heart failure with reduced ejection fraction

Age	Iron deficiency, n = 185 (%)			No iron deficiency, n = 186 (%)	Total, n = 371 (%)
	Absolute iron deficiency	Functional iron deficiency	Total iron deficiency		
≤30 years	1 (2.27%)	3 (2.13%)	4 (2.16%)	5 (2.68%)	9 (2.43%)
31–40 years	2 (4.55%)	5 (3.55%)	7 (3.78%)	15 (8.06%)	22 (5.93%)
41–50 years	8 (18.18%)	26 (18.44%)	34 (18.38%)	36 (19.35%)	70 (18.87%)
51–60 years	7 (15.91%)	41 (29.08%)	48 (25.95%)	65 (34.94%)	113 (30.46%)
>60 years	26 (59.09%)	66 (46.81%)	92 (49.73%)	65 (34.94%)	157 (42.32%)
Total	44 (23.78%)	141 (76.22%)	185 (100%)	186 (100%)	371 (100%)

**Table 2:** Gender distribution in HFrEF patients. HFrEF = heart failure with reduced ejection fraction

Sex	Iron deficient, n = 185 (%)			Iron replete, n = 186 (%)	Total, n = 371
	Absolute iron deficiency	Functional iron deficiency	Total iron deficiency		
Male	25 (56.81%)	81 (57.45%)	106 (57.30%)	149 (80.11%)	255 (68.73%)
Female	19 (43.19%)	60 (42.55%)	79 (42.70%)	37 (19.89%)	116 (31.27%)
Total	44 (100%)	141 (100%)	185 (100%)	186 (100%)	371 (100%)

**Table 3:** BMI distribution in HFrEF patients. BMI = body mass index; HFrEF = heart failure with reduced ejection fraction

BMI group	Absolute iron deficiency	Functional iron deficiency	P-value	Iron deficiency	No iron deficiency	P-value
Underweight	1 (2.27%)	1 (0.71%)	0.785	2 (1.08%)	2 (1.08%)	0.823
Normal	23 (52.27%)	73 (51.77%)		96 (51.89%)	88 (47.31%)	
Overweight	10 (22.73%)	29 (20.57%)		39 (21.08%)	46 (24.73%)	
Obese	10 (22.73%)	38 (26.95%)		48 (25.95%)	50 (26.88%)	
Total	44	141		185	186	

**Figure 2:**
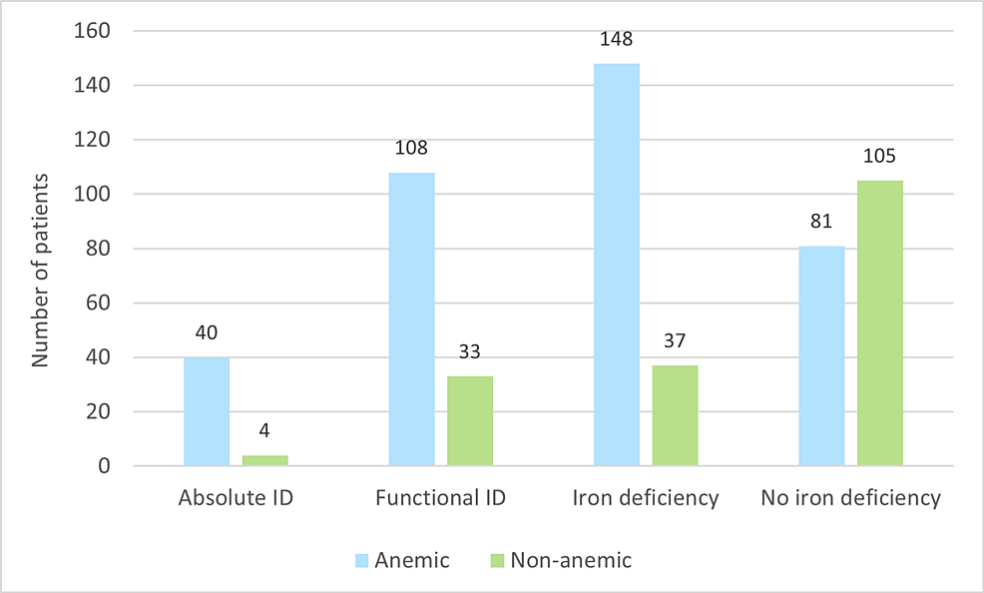
Association of ID with anemia. ID = iron deficiency

Of the 185 participants with iron deficiency, 148 (80%) presented with anemia. There was a statistically significant association of anemia with iron deficiency, as well as with absolute and functional iron deficiency (p < 0.05) (Figure 2).

**Figure 3:**
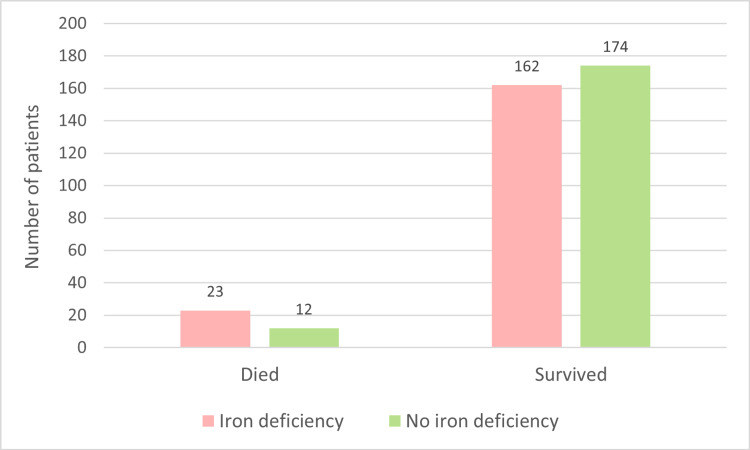
Mortality in iron deficiency.

The chi-square test was employed to examine whether a rise in mortality is associated with iron deficiency among patients diagnosed with HFrEF with a significant association (χ^2^ (1, N = 371) = 3.88, p = 0.048). Iron-deficient patients were 1.9 times more likely to die than iron-replete (relative risk = 1.9, 95% confidence interval = 0.9884 to 3.7570, p = 0.0541) (Figure 3).

**Figure 4:**
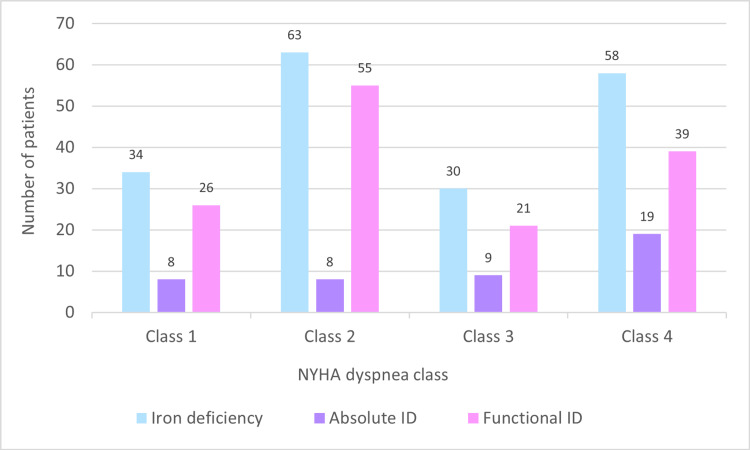
Distribution of dyspnea classes with total, absolute, and functional iron deficiency. NYHA = New York Heart Association

Results of the Spearman correlation indicated a significant large positive relationship between absolute iron deficiency and NYHA dyspnea severity, (r^2^ = 0.949, p = 0.026). No significant relationship was found between functional or total iron deficiency and dyspnea severity. Figure 4 illustrates the distribution of patients in different NYHA classes.

Although mean LVEF was less in iron-deficient patients than in non-deficient, the difference was not statistically significant (p = 0.299). Similarly, the difference between absolute and functional iron deficiency was also statistically non-significant (p = 0.0536) (Table 4).

**Table 4:** Distribution of LVEF and iron deficiency. LVEF = left ventricular ejection fraction

LVEF %	Absolute iron deficiency	Functional iron deficiency	Iron deficiency	No iron deficiency
15	0 (0.00%)	2 (1.42%)	2 (1.08%)	3 (1.61%)
20	4 (9.09%)	2 (1.42%)	6 (3.24%)	10 (5.38%)
25	7 (15.91%)	27 (19.15%)	34 (18.38%)	19 (10.22%)
30	16 (36.36%)	33 (23.40%)	49 (26.49%)	49 (26.34%)
35	9 (20.45%)	36 (25.53%)	45 (24.32%)	49 (26.34%)
40	8 (18.18%)	41 (29.08%)	49 (26.49%)	56 (30.11%)
Mean LVEF %	31.1364	32.8723	32.4595	33.0376

Participants in our study population presented with one or more symptoms, signs, and comorbidities which we considered separately.

The association of various heart failure symptoms between patients with and without iron deficiency was analyzed by the chi-square test. There was a statistically significant association between chest pain and iron deficiency (p = 0.042), as well as between fatigue/tiredness, pedal edema, orthopnea, and paroxysmal nocturnal dyspnea with absolute iron deficiency (p < 0.05) (Table 5).

**Table 5:** Association between symptoms and iron deficiency

Symptoms	Absolute iron deficiency	P-value	Functional iron deficiency	P-value	Iron deficiency	No iron deficiency	P-value
Orthopnea	20 (45.45%)	0.023	41 (29.08%)	0.728	61 (32.97%)	51 (27.42%)	0.26
Paroxysmal nocturnal dyspnea	16 (36.36%)	0.019	32 (22.70%)	0.796	48 (25.95%)	33 (17.47%)	0.6
Pedal edema	25 (56.82%)	0.005	51 (36.17%)	0.826	76 (41.08%)	61 (32.80%)	0.107
Fatigue	22 (50%)	0.001	119 (84.40%)	0.221	152 (82.16%)	148 (79.57%)	0.598
Palpitations	11 (25%)	0.425	29 (20.57%)	0.895	40 (21.62%)	35 (18.82%)	0.52
Chest pain	25 (56.82%)	0.509	80 (56.74%)	0.123	105 (56.76%)	125 (67.20%)	0.042
Syncope	1 (2.77%)	0.494	8 (5.67%)	1	9 (4.86%)	13 (6.90%)	0.511
Oliguria	3 (6.82%)	0.103	4 (2.84%)	1	7 (3.78%)	3 (1.61%)	0.22
Total	44		141		185	186	

Of the 185 participants with iron deficiency, 66 (35.68%) participants had edema and 65 (35.14%) had pallor, and the difference was statistically significant between iron-deficient and iron-replete patients. There was a statistically significant association of edema and respiratory crepts with absolute iron deficiency and of pallor with functional iron deficiency (p < 0.05) (Table 6).

**Table 6:** Association of signs and iron deficiency.

Signs	Absolute iron deficiency	P-value	Functional iron deficiency	P-value	Iron deficiency	No iron deficiency	P-value
Pallor	15 (34.09%)	0.093	50 (35.46%)	<0.001	65 (35.14%)	23 (12.37%)	<0.001
Clubbing	0 (0.00%)	1	1 (0.71%)	0.382	1 (0.54%)	0 (0.00%)	1
Edema	21 (47.73%)	0.014	45 (31.91%)	0.728	66 (35.68%)	47 (25.27%)	0.042
Icterus	0 (0.00%)	1	1 (0.71%)	1	1 (0.54%)	1 (0.54%)	1
Respiratory crept	28 (63.64%)	0.003	59 (41.84%)	0.829	87 (47.03%)	72 (38.71%)	0.116
Ascites	29 (65.91%)	0.742	82 (58.16%)	0.12	111(60%)	124 (66.67%)	0.197
Total	44		141		185	186	

A significant association was found between respiratory disease and iron deficiency (p < 0.05), between diabetes mellitus and absolute iron deficiency (p < 0.05), and between respiratory disease and functional iron deficiency (p < 0.05) (Table 7).

**Table 7:** Association between comorbidities and iron deficiency. HTN = hypertension; CAD = coronary artery disease; CVA = cerebrovascular accident; TIA = transient ischemic attack

Comorbidities	Absolute iron deficiency	P-value	Functional iron deficiency	P-value	Iron deficiency	No iron deficiency	P-value
Hypertension	30 (68.18%)	0.737	92 (65.25%)	0.911	122 (65.95%)	118 (63.44%)	0.664
Diabetes Mellitus	28 (63.64%)	0.037	64 (45.39%)	0.392	92 (49.73%)	88 (47.31%)	0.678
CAD	37 (84.09%)	0.818	120 (85.11%)	0.879	157 (84.86)	161 (86.56%)	0.659
Obesity	10 (22.73%)	0.281	27 (19.15%)	0.39	37 (20%)	25 (13.44%)	0.097
Liver disease	0 (0.00%)	1	2 (1.42%)	0.144	2 (1.08%)	0 (0.00%)	0.248
Respiratory disease	1 (2.27%)	0.712	13 (9.22%)	0.007	14 (7.57%)	5 (2.69%)	0.036
CVA (stroke, TIA)	4 (9.09%)	0.129	6 (4.26%)	1	10 (5.41%)	7 (3.76%)	0.47
Valvular heart disease	0 (0.00%)	0.607	6 (4.26%)	0.089	6 (3.24%)	3 (1.61%)	0.337
Thyroid disease	2 (4.55%)	0.753	13 (9.22%)	0.142	15 (8.11%)	10 (5.38%)	0.309
Total	44		141		185	186	

Patients with absolute iron deficiency were more likely to have raised JVP (χ^2^ (1, N = 185) = 8.71, p = 0.003) compared to functional iron deficiency patients. However, no such association was found between iron-deficient and iron-replete patients (Table 8).

**Table 8:** Association between JVP and iron deficiency. JVP = jugular venous pressure

JVP	Absolute iron deficiency	Functional iron deficiency	P-value	Iron deficiency	No iron deficiency	P-value
Raised (>4 cm)	22 (50%)	37 (26.24%)	0.003	59 (31.89%)	44 (23.65%)	0.07
Not raised (0–4 cm)	22 (50%)	104 (73.76%)		126 (68.11%)	142 (76.35%)	
Total	44	141		185	186	

Overall, 73 (39.46%) participants out of a total of 185 participants with iron deficiency had a history of coronary angiography (CAG). A statistically significant association between a history of CAG and iron deficiency was present (χ^2^ (1, N = 371) = 10.06, p = 0.002) as well as between a history of CAG with absolute and functional iron deficiency (χ^2^ (1, N = 185) = 5.05, p = 0.0246 (Table 9).

**Table 9:** Association of a history of CAG and iron deficiency. CAG = coronary angiography

CAG	Absolute iron deficiency	Functional iron deficiency	P-value	Iron deficiency	No iron deficiency	P-value
Yes	11 (25%)	62 (43.97%)	0.0246	73 (39.46%)	104 (55.91%)	0.002
No	33 (75%)	79 (56.03%)		112 (60.54%)	82 (44.09%)	
Total	44	141		185	186	

A statistically significant difference was present between tobacco and smoking addiction with iron deficiency (p < 0.05) (Table 10).

**Table 10:** Association of addictions with iron deficiency.

Addictions	Iron deficiency	No iron deficiency	P-value
Tobacco	72 (38.92%)	103 (55.38%)	0.001
Smoking	64 (34.59%)	87 (46.77%)	0.017
Alcohol	46 (24.86%)	60 (32.26%)	0.114

A comprehensive analysis of CBC, serum iron studies, LFT, RFT, TFT, blood glucose tests, lipid profile, and CRP was conducted, as outlined in Table 11. The results revealed statistically significant differences between patients with and without iron deficiency in hemoglobin, serum iron, serum ferritin, TSAT, and total iron-binding capacity (TIBC). Additionally, significant differences were noted between absolute and functional iron deficiency concerning hemoglobin, blood urea nitrogen, serum glutamic oxaloacetic transaminase, serum glutamic pyruvic transaminase, total protein, serum albumin, and serum ferritin levels (p < 0.05).

**Table 11:** Comparison of blood parameters with iron deficiency. CBC = complete blood count; Hb = hemoglobin; WBC = white blood cell; TSAT = transferrin saturation; TIBC = total iron-binding capacity; RFT = renal function test; BUN = blood urea nitrogen; SGPT = serum glutamic pyruvic transaminase; SGOT = serum glutamic oxaloacetic transaminase; LFT = liver function test; TFT = thyroid function test; TSH = thyroid-stimulating hormone; FBS = fasting blood sugar; RBS = random blood sugar; HbA1C = glycated hemoglobin; HDL = high-density lipoprotein; LDL = low-density lipoprotein; VLDL = very low-density lipoprotein

	Parameter	Normal range and unit	Absolute iron deficiency, mean (±SD)	Functional iron deficiency, mean (±SD)	P-value	Iron deficiency, mean (±SD)	No iron deficiency, mean (±SD)	P-value
CBC	Hb	Males: 14–17 g/dL Females: 12–15 g/dL	9.8 (±2)	11.1 (±1.9)	<0.001	10.8 (±2.0)	13.0 (±2.3)	0.0001
	WBC	4,500–11,000/mm^3^	10,120.5 (±3,974.7)	10,246 (±4,479.5)	0.929	10,216.1 (±4,354.6)	10,129.4 (±3,853.3)	0.839
	Platelet	1.5–4 lakhs/mm^3^	2.4 (±1)	2.7 (±0.8)	0.072	2.6 (±0.9)	2.6 (±0.9)	0.778
Serum iron studies	Serum iron	60–170 µg/dL	57.1 (±99.6)	51.2 (±15.2)	0.492	52.6 (±50.1)	69.0 (±22.9)	<0.001
	Serum ferritin	Males: 12-300 ng/mL Females: 12–150 ng/mL	76.4 (±26.8)	154.7 (±49.5)	0.0001	136.1 (±56.1)	313.2 (±560.6)	<0.001
	TSAT	15–50%	14.1 (±12.3)	15.2 (±3.1)	0.331	14.9 (±6.6)	38.2 (±13.2)	<0.001
	TIBC	240-450 mcg/dL	293.1 (±63)	284.3 (±68.8)	0.448	286.4(± 67.4)	225.1(± 58.7)	< 0.001
RFT	BUN	6–24 mg/dL	23.9 (±12.4)	19.2 (±9.6)	0.033	20.3 (±10.5)	19.9 (±14.1)	0.765
	Creatinine	0.6–1.3 mg/dL	1.5 (±0.7)	1.3 (±0.4)	0.101	1.4 (±0.5)	1.4 (±0.6)	0.809
LFT	SGPT	7–56 U/L	38.6 (±32.7)	34.2 (±35.4)	0.0001	35.2 (±34.8)	36.7 (±43.3)	0.711
	SGOT	8–45 U/L	41.2 (±27.7)	44.4 (±42.6)	0.0001	43.6 (±39.5)	49.4 (±70.4)	0.336
	Total protein	6–8.3 g/dL	5.5 (±2.3)	6.4 (±1.8)	0.0001	6.2 (±1.9)	6.6 (±5.1)	0.25
	Serum albumin	3.4–5.4 g/dL	2.9 (±1.4)	3.5 (±1)	0.0001	3.4 (±1.2)	3.7 (±2.8)	0.175
TFT	T3	80–220 ng/dL	94.6 (±29.7)	91.3 (±27.5)	0.385	92.1 (±28.0)	90.7 (±24.7)	0.625
	T4	5–12 µg/dL	8.6 (±3.5)	7.9 (±3.4)	0.932	8.0 (±3.5)	9.4 (±11.2)	0.114
	TSH	0.4–4 mIU/L	4.1 (±2.4)	3.6 (±2.6)	0.384	3.7 (±2.5)	3.8 (±2.5)	0.651
Blood glucose tests	FBS	70–110 mg/dL	153.1 (±69.1)	140.5 (±46.2)	0.136	143.5 (±52.6)	139.8 (±55.6)	0.513
	RBS	<140 mg/dL	152.2 (±65.7)	142.3 (±48.9)	0.196	144.6 (±53.4)	141.1 (±48.3)	0.502
	HbA1c	<5.7%	7.6 (±2.2)	7.2 (±1.8)	0.134	7.3 (±1.9)	7.1 (±1.7)	0.31
Lipid profile	Cholesterol	<170 mg/dL	153.6 (±44.6)	156.2 (±46.5)	0.464	155.5 (±46.0)	161.8 (±51.6)	0.222
	Triglyceride	<150 mg/dL	133.7 (±57.4)	130.2 (±98.7)	0.980	131.0 (±90.5)	135.8 (±69.9)	0.57
	HDL	>45 mg/dL	42.5 (±30.2)	36.4 (±9.4)	0.162	37.8 (±16.7)	39.5 (±21.4)	0.407
	LDL	<100 mg/dL	89.8 (±40.4)	92.5 (±36.4)	0.724	91.8 (±37.3)	91.7 (±40.4)	0.969
	VLDL	2–30 mg/dL	28.0 (±20.5)	26.5 (±18.7)	0.426	26.9 (±19.1)	24.9 (±17.3)	0.306
Serum inflammatory marker	CRP	<10 mg/L	24.7 (±31.1)	19.1 (±29.7)	0.286	20.5 (±30.1)	16.5 (±24.9)	0.166

## Discussion

Iron deficiency, a health-related condition with insufficient iron availability, can occur with or without anemia [4]. Causes of iron deficiency in heart failure are multifactorial, including chronic inflammation, reduced iron intake, decreased gastrointestinal (GI) iron absorption due to edema, and increased GI blood loss (partially from antiplatelet and anticoagulant drugs) [16]. Exercise intolerance and fatigue are defining features of heart failure patients. Studies reveal that iron deficiency, even before anemia onset, can be severe in heart failure patients, worsening the disease and negatively impacting symptoms, QoL, exercise capacity, and clinical outcomes. Iron deficiency in heart failure is associated with an increased risk of mortality and doubles the risk of hospitalization (relative risk = 2.23) [17].

Iron exists in circulating and stored forms, with stored iron being mobilizable or immobilizable. The liver-secreted protein, hepcidin, regulates ferroportin activity, an iron exporter in various cell types, including gut mucosal cells, hepatocytes, and macrophages [18]. The binding of hepcidin to ferroportin leads to lysosomal destruction, reducing ferroportin levels and inhibiting iron release [18]. In heart failure, elevated hepcidin levels hinder iron mobilization, despite sufficient iron stores, impacting metabolic functions and hematopoiesis. The chronic inflammatory state in heart failure patients can sequester iron in macrophages, limiting its availability for erythropoiesis. Iron deficiency is categorized into absolute and functional iron deficiency. Anemia occurs only when iron deficiency becomes severe enough to hinder erythropoiesis and decrease hemoglobin production. Absolute iron deficiency is identified when serum ferritin is <100, while functional iron deficiency is characterized by serum ferritin between 100 and 299 and TSAT <20% [4]. Iron deficiency can result from insufficient absorption or chronic blood loss, leading to low iron storage. Low circulating iron and ferritin levels indicate absolute iron deficiency, while functional ID, caused by inflammation, shows low iron but normal or elevated ferritin concentrations. In both cases, limited iron availability for erythropoiesis leads to eventual anemia. In our study with 371 participants, 12% had absolute iron deficiency, 38% had functional iron deficiency, and, overall, 50% had iron deficiency.

As people age, their hearts undergo structural and functional changes, increasing the risk of heart failure. Common changes include increased stiffness in the heart muscle, decreased efficiency in the heart’s electrical system, and a reduction in the heart’s ability to pump blood. Older adults are at a higher risk due to accompanying health conditions such as high blood pressure, diabetes, and coronary artery disease (CAD) [19]. In our study, participants’ ages ranged from 23 to 89 years, with an average age of 57.53 (±11.85) years.

Of the 185 iron-deficient participants, 57.5% were male and 42.5% female. The background for this observation is unclear but may be linked to gender-specific differences in the regulation of iron status by hormones such as estrogens [20] and testosterone [21] or altered immune regulation and inflammatory profiles between men and women based on genetic and hormonal factors [22].

Obesity is a recognized risk factor for iron deficiency, likely due to heightened inflammation and hepcidin levels, which hinder iron absorption. Iron deficiency is linked to HFrEF, impacting oxygen transport to tissues, including the heart. In a 2017 study published in the Journal of the American College of Cardiology, obese HFrEF patients showed a higher prevalence of iron deficiency, independently associated with worse outcomes, including increased hospitalization and mortality [23]. In our study, nearly 50% of obese patients were iron deficient, with 79% having functional iron deficiency and 20% having absolute iron deficiency. The intricate relationship between obesity, iron deficiency, and HFrEF, while not fully understood, appears to contribute to the development and progression of heart failure.

In this study, 61% of all participants and 80% of those with iron deficiency had anemia, showing a significant association between iron deficiency and anemia (p < 0.05). The connection between iron deficiency and anemia in HFrEF involves complex factors such as inflammation, renal dysfunction, and impaired erythropoiesis. Anemia in chronic heart failure often lacks a clear etiology, with inflammation emerging as a central component. Patients with advanced heart failure exhibit immune activation and elevated cytokine levels [24]. Inflammation induces cytokines and the master regulator of iron homeostasis, hepcidin, impeding dietary iron uptake and macrophage iron release [24]. Hepcidin interacts with ferroportin-1, leading to its internalization, limiting duodenal iron absorption and macrophage iron release [24]. Consequently, inflammation retains iron in the mononuclear phagocyte system, resulting in low circulating iron levels and increased ferritin concentrations [25]. Originally a defense mechanism, these iron homeostasis changes restrict microbial nutrient availability during inflammation [25]. Iron deficiency without anemia in heart failure impacts functional parameters such as oxygen consumption and myocardial contractility. This may be linked, in part, to impaired mitochondrial function as a consequence of iron deficiency, as iron stimulates Krebs cycle activity and oxidative phosphorylation [26]. Both iron deficiency and anemia independently predict adverse outcomes in HFrEF patients, including increased hospitalization rates and mortality [27].

In our study, out of the 185 patients with iron deficiency, 12.42% succumbed to death, while among 186 patients without iron deficiency, 6.45% dies. A statistically significant association between iron deficiency and mortality was observed in patients with HFrEF. The findings of Beverborg et al. align with our findings, indicating a strong association between iron deficiency in HFrEF and increased mortality and heart failure hospitalization rates [27]. Iron deficiency in HFrEF is linked to elevated mortality through various mechanisms, including impaired oxygen delivery, leading to tissue hypoxia and damage, and increased oxidative stress contributing to tissue damage and inflammation, which are known drivers of heart failure progression and mortality. Additionally, the activation of inflammatory pathways, marked by increased proinflammatory cytokines and acute-phase reactants, highlights the role of chronic inflammation as a hallmark in heart failure progression and adverse outcomes [24].

Dyspnea, or difficulty breathing, is a common symptom of HFrEF. The root cause of dyspnea in HFrEF is primarily pulmonary congestion, resulting from the heart’s inefficient pumping. HFrEF involves impaired pumping function, leading to decreased blood ejected per heartbeat (reduced ejection fraction). This diminished blood flow can result in fluid buildup in the lungs, causing pulmonary congestion. The elevated pressure and fluid accumulation make breathing challenging, particularly during physical activity or while lying down. Factors contributing to dyspnea in HFrEF include reduced oxygen delivery to tissues due to decreased cardiac output, an increased respiratory rate from compensatory mechanisms such as hyperventilation to maintain oxygenation, and lung gas exchange abnormalities leading to elevated carbon dioxide and reduced oxygen levels in the blood. Ultimately, the primary cause of dyspnea in HFrEF is pulmonary congestion due to impaired cardiac function [28]. In our study, we observed a significant positive relationship between absolute iron deficiency and the severity of dyspnea assessed on the NYHA dyspnea scale (r^2^ = 0.949, p = 0.026).

Iron, a key component of hemoglobin, plays a vital role in oxygen transport. Iron deficiency can result in fewer red blood cells, reducing oxygen delivery to the heart. This prompts the heart to work harder, potentially decreasing LVEF. Additionally, iron deficiency has been linked to impaired mitochondrial function, reducing the energy available for the heart to pump blood and further impacting LVEF [29]. In our study, the mean LVEF in iron-deficient patients was 32.45% compared to 33.03% in non-deficient individuals; however, this difference was statistically insignificant. LVEF, a crucial measure of heart function, represents the percentage of blood pumped out of the left ventricle with each heartbeat. Although iron deficiency can induce changes in the heart, impacting LVEF, our study did not establish a significant difference in LVEF between iron-deficient and non-deficient groups.

Out of the 185 participants with iron deficiency, 60% had ascites, 47.03% had respiratory crepitations, 35.68% had edema, and 35.14% had pallor. Pallor and pedal edema are notable clinical features in individuals with iron deficiency and HFrEF. Iron deficiency can lower hemoglobin levels, causing pallor due to decreased oxygen-carrying capacity of the blood. Additionally, iron deficiency may contribute to weakness, fatigue, and shortness of breath, which are common symptoms of heart failure. Pedal edema, the accumulation of fluid in the feet and ankles, is a common symptom of heart failure, exacerbated by iron deficiency due to potential decreases in serum albumin levels [1]. Therefore, the presence of pallor and pedal edema in patients with iron deficiency and HFrEF suggests a need for further evaluation and treatment. Iron replacement therapy and heart failure management may help alleviate these symptoms and improve outcomes.

Among the 185 patients with iron deficiency, 84.86% had coronary artery disease, followed by hypertension in 65.95%, diabetes mellitus in 49.73%, obesity in 20%, respiratory disease in 7.57%, thyroid disease in 8.11%, and others. HFrEF often coexists with various comorbidities that contribute to iron deficiency. Common comorbidities associated with iron deficiency in HFrEF patients include chronic kidney disease, diabetes mellitus, chronic obstructive pulmonary disease (COPD), and coronary artery disease. CKD, prevalent in HFrEF, independently predicts iron deficiency through mechanisms such as reduced erythropoietin production, impaired iron absorption, and increased blood loss due to coagulopathy [23]. Diabetes mellitus, linked to HFrEF, increases iron deficiency risk due to chronic inflammation and oxidative stress. It can also lead to diabetic nephropathy, a common cause of CKD. COPD, associated with HFrEF, heightens iron deficiency risk through chronic inflammation and increased blood loss during exacerbations. CAD, another common comorbidity in HFrEF, may induce iron deficiency through chronic blood loss from the GI tract or other sources [10]. Overall, comorbidities such as CKD, diabetes mellitus, COPD, and CAD contribute to iron deficiency in HFrEF patients through various mechanisms.

In our study, 39.46% of the 185 participants with iron deficiency had a history of CAG. Patients with CAD often undergo CAG for diagnosis and management. However, those with CAD undergoing CAG face an increased risk of GI bleeding due to anticoagulant and antiplatelet medications and procedural stress, potentially leading to chronic blood loss and subsequent iron deficiency [10].

Substance abuse can lead to health issues, including malnutrition and anemia, contributing to iron deficiency. It can also damage the heart muscle, leading to HFrEF. A 2016 study in the Journal of Cardiac Failure found higher iron deficiency prevalence in HFrEF patients with a history of substance abuse, associated with more severe heart failure symptoms, lower exercise tolerance, and poorer QoL [30]. Our study also identified a statistically significant association between tobacco/smoking addiction and iron deficiency (p < 0.05).

In this study, among patients with iron deficiency, the average serum iron value was 52.6 (±50.1) μmol/L, serum ferritin averaged 136.1 (±56.1) μg/L, TSAT averaged 14.9 (±6.6)%, and TIBC averaged 286.4 (±67.4) mg/dL. Iron deficiency results in decreased serum iron levels due to insufficient iron for hemoglobin and other iron-containing proteins. Serum ferritin, reflecting body iron stores, is typically reduced in iron deficiency as stores deplete. TSAT measures the percentage of transferrin molecules saturated with iron, often decreased in iron-deficient patients. TIBC, indicating the amount of iron that can bind to transferrin, is typically elevated in iron deficiency due to compensatory mechanisms, leading to higher TIBC values [31].

The study’s identification of significant differences in health outcomes related to iron deficiency in HFrEF patients suggests a potential need for targeted screening programs and early interventions. This information could shape clinical guidelines, influencing the incorporation of iron status monitoring in HFrEF management. Public awareness campaigns might be essential to educate both healthcare providers and individuals about the implications of iron deficiency in this context.

This study has a few limitations. Resource constraints prohibited the assessment of anemia causes other than iron deficiency. The research size and site restrictions hinder the generalization of results to the entire community. The cause-and-effect relationship may not be accurately depicted in such a small sample. Given that the study was conducted in an urban tertiary care hospital, the data primarily reflects patients in urban areas. Consequently, the generalizability of the results is limited for rural areas. A larger and more diverse sample from various locations could provide a more reliable outcome.

## Conclusions

The pervasive prevalence of iron deficiency in patients with HFrEF makes it a noteworthy concern. This condition is not only significantly associated with increased mortality in HFrEF patients but also stands out as a common comorbidity, independent of the presence of anemia. The correlation of absolute iron deficiency with increased dyspnea severity adds to the complexity of its impact on the clinical profile of these patients. The cascading effects, including reduced hemoglobin synthesis, impaired oxygen delivery, heightened oxidative stress, and activation of inflammatory pathways, contribute substantially to heart failure progression and adverse outcomes. Current literature emphasizes the adverse impact of iron deficiency on exercise capacity, health-related QoL, and mortality in heart failure patients, prompting interest in interventions to correct iron deficiency. Nevertheless, further research is imperative to refine strategies for optimal identification and management of iron deficiency in HFrEF patients, elucidating the intricate mechanisms that underlie its association with adverse outcomes in this population.
